# Personal Care of the Teeth

**Published:** 1900-02

**Authors:** A. H. Thompson


ARTICLE II.

### PERSONAL CARE OF THE TEETH.

A. H. THOMPSON, D. D. S.

It ought not to be necessary to urge that as the mouth, the oral cavity, is the gateway, the vestibule of the alimentary canal, wherein the food is received and partially prepared for digestion that is essential to the maintenance of life, that this cavity should be kept in a pure, clean and wholesome condition. But as it is seldom kept in such a condition, we cannot wonder that there should be so much disease in the human system, but rather that there should be so little. The mouth is too often neglected to such an extent that it is a perfect pool of filth, containing organic matter that is continually fermenting and putrefying, and vitiating not only the food and liquids that pass through the mouth, but emitting gases also which contaminate the air breathed into the lungs. Carious teeth and diseased gums also exude putrefactive secretions and gases, which pass into the stomach and lungs, and undoubtedly contribute to disease. But good Mother Nature is long-suffering and energetic, and carries well the burden of resisting the infection introduced every day by means of the food and water and air passing through this filthy gateway. There is little doubt but that the mouth, kept clean, and antiseptically clean, would do much to counteract, if not prevent, many general as well as local diseases of septic nature. This can be readily accomplished by careful attention to simple hygienic measures, whose efficiency is well understood, and which can be readily employed.

One of the first elements in the hygiene of the mouth is the proper care of the teeth, with a view to their preservation in a cleanly and wholesome condition. As is well known, the teeth of man, especially of the higher races, are in a degenerate condition as regards their structural integrity, and that, in consequence, they fall ready victims to the attacks of destructive diseases. Without artificial resources for their preservation, the teeth of civilized man would be lost at an early age in the average individual. As is well known, when the teeth are lost, or are inefficient for the duty of masticating the food properly, that it cannot be well digested, and that this leads to dyspepsia and all the evils that follow in the train of this distressing affliction. Therefore, physiologists say that the teeth belong to the alimentary system, and bear an important part in the digestive process. It is necessary that the food should not only be well masticated, but that it should be well mixed with the saliva by the same process.

The first step in the process of putting the teeth and mouth in a healthy condition is to have all cavities of decay filled, and the teeth carefully cleaned of all deposits about the gums, and carefully polished by a skillful dentist. Little can be done by the individual until the teeth are well cleaned with proper instruments, and all deposits of stain and calculus removed. Then they are in condition for the individual to take proper care of, and not until then. The hard calculus that accumulates under the gums and makes them diseased cannot be removed by the brush, but requires proper instruments.

The bacteria of decay collect in various places, especially in depressions and imperfections of the enamel where they are not easily disturbed, and become the nucleus of decay. Particles of food accumulating and decomposing also become centers of disease. Therefore, these accumulations should be removed, and the formation of incipient decay prevented as much as can be. The enamel should be kept polished, and the lodgment and ill-effects of deleterious material thereby rendered more difficult. To accomplish these ends, the most effective means is the use of a properly shaped tooth-brush and a polishing dentifrice, with an antiseptic wash. This is an operation that is simple, but is effective, although it is imperfectly performed by the average individual. Perhaps its very simplicity contributes to its careless performance. The proper kind and shape of tooth-brush has been much discussed, but there is no one kind or quality that is suited to the needs of every individual. For instance, the person whose teeth have rough enamel from long neglect or disease should use a flat, hard brush with sharp dentifrice until the enamel is well polished. But the next person, who, from long years of hard brushing, has well-polished enamel, with perhaps a tendency to receding of the gums, would have his teeth injured by this means. He would require a softer brush with a less sharp dentifrice. A very soft brush should be used only by persons with diseased gums until these are well, or during an illness, when the mouth and gums are very irritable. In a general way, the best form of brush is that which is curved from heel to point to fit the curve of the dental arch, and has transverse rows of bristles of unequal height to cleanse between the teeth.

The proper motion to be given the brush is also an important matter. The person with rough enamel should use the brush hard in every direction, but the one with well-polished enamel, with the gums receding from previous hard brushing, should carefully avoid the cross-motion and use the vertical movement; that is, the upper teeth should be brushed downward and the lower upward, away from the gums both above and below. The motion will cleanse the spaces between the teeth and prevent wear upon the prominent portions of the enamel and the gums. To accomplish this motion, which is a little difficult at first, the hand is rotated, thereby causing the bristles to pass from the gums toward the occlusal surfaces of the teeth, the ridges on the brush passing between the teeth and cleaning these surfaces thoroughly. The lingual surfaces are brushed upwards also as much as may be and the grinding faces transversely. The proper method of brushing the teeth is but imperfectly understood by the laity, and dentists do not, as a rule, give their patients sufficiently full instructions in regard to this important matter.

The next important matter is that of a proper dentifrice. This is supplied in the form of powders, tablets, pastes, soaps, etc. But the form of the preparation is of little consequence, nor is the color or flavor, except that it should be attractive and pleasant to use. A pleasant flavor is likely to be an inducement to children and weak-willed persons to use a dentifrice, and is, therefore, useful. A good dentrifrice is composed of polishing powders, antacids, antiseptics, and coloring matter and flavor. Soap is an ingredient that is more or less useful, but is liable to discolor the teeth by too prolonged use. Astringent gums are sometimes added, but are liable to be injurious to the gums by continued use. Patent dentifrices are, as a rule, to be avoided, for they are likely to contain chemical ingredients which whiten the enamel, but which will also corrode and injure it. Different persons require different qualities of dentifrice. The person with rough enamel needs a sharp powder, but this would be injurious to the person with smooth and well-polished enamel, for it would be too wearing. A softer powder is best for the latter. The occasional use of soap is permissible if desirable, but it does not take the place of a polishing dentifrice. Proper dentifrices should be directed by the dentist as suited to the needs of each individual.

The proper time to employ the dentifrice is in the morning, to remove the accumulations of mucus and stain of the night. The mouth should then be put in a clean and wholesome condition also, before introducing food into it, as a matter of decency. The dentifrice should be used plentifully by those with rough and stained teeth and where there is much disposition to the rapid accumulation of deposits and stain, but those with polished teeth, which are habitually clean because polished, should use the powder sparingly. There is much difference as to the rapidity of the accumulation of stain and tartar, and the amount of brushing and powder used must be governed accordingly.

The employment of an antiseptic wash is the next necessity in the proper care of teeth. As the mouth is a perfect aquarium of bacteria and germs which give rise to fermentation and putrefaction, it is necessary that a proper antiseptic wash should be employed in the cleaning of this cavity. For this purpose the water that is used should be as warm as can be borne by the mouth for its solvent and cleansing powers, to which should be added two teaspoonfuls to the glass of a proper antiseptic preparation. For this purpose the preparations known as listerine, euthymol, borolyptol, pasteurine, sanitol, etc., are the most agreeable and effective, but other antiseptic washes will answer as good a purpose. This wash should be used in the morning with the brush and powder and on retiring at night without the powder. It is during the quiet hours of the night, when the movements of the mouth are suspended, when the salivary glands rest from their duty of irrigating the mouth and particles of food that remain about the teeth to ferment and decompose, that the antiseptic wash is most useful and should be freely employed. Removing particles of food from between the teeth with the toothpick or waxed floss silk after meals is a necessary hygienic measure, that it may not be retained to decompose and become the seat of caries. The ordinary wood toothpick which is in such general use is more or less injurious, as it is liable to leave splinters between the teeth, causing irritation and disease of the gums. Persistent use of the wood toothpick also pushes up the gum and wears the teeth at the neck. The quil pick is better, but it is inconvenient and cannot be kept clean. A cheap toothpick that is tough and smooth is much needed. For ladies, of course, a toothpick is inadmissible, but they can use waxed floss silk, which is safer and more effectual for cleaning between the teeth.

Brushing the teeth after every meal is questionable, as it is apt to be hurried and hard on exposed surfaces and incomplete in others. Chewing gum after meals is better, as this cleanses the teeth and stimulates the flow of saliva, which passes into the stomach and aids digestion. This is especially desirable for children, but after meals only, as gum chewing is injurious at other times by exhausting the saliva, so that the mouth is deprived of this valuable digestive fluid at meal time.

Brushing as a means of cleaning the mouth is of advantage not only for the teeth, but for the gums, tongue, cheeks and fauces, which should be cleansed with the brush and the antiseptic wash as well. The brush should be passed over the gums, the spaces between the gums and cheeks and lips, the inside gum, the tongue, the sublingual space and the palate and fauces as far down as may be bearable. It will be found that the health of these surfaces will be much benefited by the removal of the accumulations that ferment and become offensive. When there is any catarrhal tendency, these accumulations become dangerous by infecting the air that is breathed into the lungs. Of course, at such a time there should be proper cleansing of the nasal passages and pharynx, but this should be supplemented by cleaning the mouth and fauces as well.

The teeth of children should have special care, that they may be kept in healthful condition, and the mouth should be kept clean and wholesome. They should be taught at an early age to use the brush and dentifrice and be made to feel that it is a reflection upon their personal habits of cleanliness to neglec it, as it is to have dirty faces; that a dirty mouth is as bad as a dirty face. It wlll soon become a matter of pride with them to keep the teeth clean. The black stain that accumulates about the margins of the gums on the teeth of children is best removed with a flat pointed stick and sharp tooth powder. The stain is common to children and does no harm beyond its bad appearance. Of course the antiseptic wash should be freely employed by children, cleaning and washing the mouth and throat with it morning and evening. If some should happen to be swalled, it would do no harm. Small brushes are made for children which are easily used in their small mouths.

During pregnancy and nursing the teeth require special care, for, as is well known even by the laity, the teeth undergo rapid decay during this critical period. It was formerly supposed that during this period the lime-salts were taken from the teeth and bones of the mother to form the osseous structures of the child, and that in consequence of this deprivation her teeth fell the ready victims of rapid decay. Recent investigations have demonstrated the fact, however, thac the physical condition of the dental tissues are much the same during pregnancy as at other times, so tnat the old theory of weakened resistance is not tenable. We know now that the rapid decay of the teeth at this time is due to the peculiar composition of the oral fluids, which are acid and corrosive. It is well known that the entire physiological system of the woman is revolutionized at this time, and that her functions are greatly modified. Amongst other peculiarities is the production and prevalence, to a greater or less extent in the fluids of the body, of lactic acid, the acid of milk. This is present strongly in the fluids of the mouth, and lactic acid is most destructive to the teeth, on account of its affinity for their lime-salts. Indeed, it has been found by investigation that lactic acid is a most destructive agent created by the bacteria of decay. To counteract this destructive agent, therefore, the logical thing to do is to neutralize it and render it inert, if possible. For this purpose active antacids, such as chalk or magnesia, are employed during the entire period of gestation and lactation; prepared chalk should be packed about the teeth and left during the night, when the most destruction of tooth-tissue takes place, on account of the more active action of the acid during the hours of rest.

This will, to a large degree, counteract and neutralize the active effects of the acid condition and stop destruction of the teeth. Milk of magnesia is excellent for the purpose, as it clings to the teeth and forms a coating over the enamel and remains strongly alkaline. A strong solution of lime-water should also be employed during the waking hours at intervals, especially when the teeth feel rough and on edge. This special care will do much to counteract the acid condition of the mouth during this critical period and preserve the teeth, The dentist learns to dread the appearance of a patient who has just been through a pregnancy, as he is pretty sure to find that there is extensive decay, and that most of the dental operations in the mouth have been undermined by decay.

With persons who have soft or a more or less habitually acid condition of the mouth, the antacid treatment is often beneficial; especially is this the case during adolescence, when there is general weakness of the tissues, owing to the rapid growth and the changes of puberty. The teeth are still incomplete as to density, and special care will often tide over these critical years. As the teeth harden with advancement to puberty, they become more resisted and the fluids of the mouth are less corrosive. Some forms of dyspepsia are also destructive of the teeth, and in some forms of illness, as typhoid fever, the injury to the teeth on account of vitiated oral fluids is often very marked. Acid conditions prevail in many kinds of ill health which induce decay, but special care or special conditions will require special prescriptions, so that general directions can not be made of much value for such cases.—*The Stomatologist.*

